# Supplementary material for: Are carbon emissions trading and green financial instruments synergistic? -Comprehensive quantitative research based on content analysis
Source: PLoS One. 2024 Mar 7;19(3):e0298601. doi: 10.1371/journal.pone.0298601 (PMC10919601; doi:10.1371/journal.pone.0298601)
Supplement: S1 Data — (ZIP) [file pone.0298601.s001.zip › Data/地级市Z_F工作报告.zip!/─■╧─/╬Γ╓╥╩╨/└┤╫╘╣1⁄2╓┌║┼í╛┬φ┐╦╩2╛▌═°í┐_2004.docx]

各位代表：

现在，我代表市人民政府，向大会作政府工作报告，请予审议，并请市政协各位委员和其他列席人员提出意见。

一、过去五年工作的回顾

市一届人大一次会议也就是撤地设市以来的五年，是吴忠跨世纪发展、各项事业突飞猛进的重要时期。五年来，在自治区党委、政府和市委的正确领导下，全市各族人民高举邓小平理论伟大旗帜，认真实践“三个代表”重要思想，与时俱进，开拓创新，全面推进改革开放和现代化建设，胜利完成了一届人大一次会议确定的各项目标任务。全市呈现出经济繁荣、社会进步、民族团结、政治稳定的良好局面。

(一)国民经济快速发展，经济实力明显增强。2003年，全市国内生产总值预计达到120亿元，比1998年增加59亿元，年均增长12.1%，人均6521元。地方财政收入达到7.1亿元，年均递增 13.1%。经济结构渐趋合理，三次产业协调发展。农业转型步伐加快，产业化经营取得突破性进展，“三白一红一绿”特色产业不断发展壮大。畜牧业在农业总产值中的比重达49.1%。工业经济大幅攀升，持续保持了总产值、增加值、销售收入、利润“四个同步增长”，总产值年均递增21.1%。地方工业实现利税年均递增19%，对经济增长的贡献率达到60%，比1998年提高了24个百分点。非公有制经济增加值占国内生产总值的比重达到22.5%，提高了8个百分点。第三产业蓬勃发展，增加值达到32亿元，年均增长10%。社会消费品零售总额达28亿元,年均增长7.5%。

(二)基础设施建设步伐加快，城乡面貌显著改观。全社会固定资产投资累计完成221亿元，是前五年的1.61倍。实施了沙坡头水利枢纽、苦水河治理、香山治洪等一批重点工程。配合自治区建成了古王、石中高速公路，新建了吴惠、吴灵、吴青、盐兴等公路。全市公路总里程达5920公里，比1998年增加2670公里。加快了城乡电网改造和公众电信网、广播电视网建设，固定电话和移动电话用户分别达21万户和27.1万户，比1998年增长1.8倍和6.1倍。城市建设累计投资133.5亿元，建成区面积由29.7平方公里扩大到64.6平方公里，城市化率达到30%，比1998年提高了9个百分点，其中，吴忠市区建成区面积由1998年的7.6平方公里扩展到16平方公里。城市集中供暖、供水、排污等基础设施大为改观，城市人均公共绿地由1998年的0.48平方米提高到2.1平方米，绿化覆盖率达10.5%，其中，吴忠市区由0.4平方米提高到3.6平方米，绿化覆盖率达10.8%。实施了盛元广场、“民居计划”等一批新建工程，完成新区开发1.5平方公里。同时，各县(市、区)城市建设也取得了实质性突破，城市面貌发生了前所未有的变化，城市的集聚效应和辐射功能进一步增强。

(三)各项改革稳步推进，对外开放取得新突破。国有企业改革不断深化，盘活企业63家，盘活存量资产近10亿元，改制面达94.1%。第二轮土地承包顺利完成，农村税费改革取得明显成效，农民人均负担由73.35元减少到45.06元。行政机构改革和区划调整稳步推进，市县乡行政编制减少20%，乡镇撤并率达40.63%。全面推行政务公开，政府部门和窗口单位普遍实行公开办事制度、收费公示制度和服务承诺制度，清理取消审批事项207项。成功举办了第十三届西部商品交易会、美食节等大型活动，进一步加强了对外交流与合作，五年累计引进各类项目330个，概算总投资49.5亿元，实际到位资金17.75亿元，实际利用外资1.4亿美元。

(四)社会事业全面进步，人民生活稳步改善。大力实施“科教兴市”战略，全面推进素质教育，山区基本实现“普初”目标，川区“两基”向较高层次迈进。积极扩大高中阶段教育，兴建了吴忠高级中学；努力发展高等职业技术教育，成功组建了职业技术学院。文化体育事业健康发展，城乡人民文化生活日益丰富和活跃。人口与计划生育工作水平稳步提高，人口增长得到有效控制。卫生事业不断进步，医疗基础设施得到改善，“防治非典”工作取得实质性成效。环境保护和污染源治理力度加大，环境质量持续好转。生态建设取得明显成效，川区实现二代林网化，山区草原全面禁牧。扶贫工作扎实推进，社会保障体系逐步完善，就业和再就业工作得到加强，五年内通过各种途径安置下岗职工再就业6万人，城镇登记失业率控制在了4%以内。实现劳务输出40万人次，为1.6万名企业退休职工发放养老金4.2亿元，为1.2万名失业人员发放失业保险金5600多万元。民族宗教、司法、民政、气象、地震等工作不断加强，妇女、儿童、老龄、残疾人事业得到重视和发展。城乡居民收入稳步增长，生活水平日益提高。2003年，城镇居民人均可支配收入达到6000元，农民人均纯收入达到2600元，分别比1998年增长55.1%和22.6%。城乡居民储蓄存款余额达到106亿元，年均增长10.5%，城镇居民和农民人均住房面积达到19.7平方米和25平方米，分别比1998年增加了7平方米和6平方米。

(五)精神文明建设、民主法制建设和政府自身建设不断加强。深入开展了“三讲”、“三个代表”重要思想学教活动和形式多样的精神文明创建活动，大力弘扬金占林、马英等先进典型的时代精神，人民群众的精神风貌发生了深刻变化，建设经济强市的热情不断迸发。“双拥”及“军民共建”活动深入开展，军政、军民团结与国防后备建设进一步加强。坚持依法治市，推进依法行政，深入开展全民普法教育，基层民主政治建设不断加强，村民自治和社区居民自治有序推进。狠抓社会治安综合治理和安全生产工作，严厉打击各种违法犯罪活动和“法轮功”等邪教组织，社会治安秩序逐步好转。健全落实信访工作制度，妥善处理人民内部矛盾，有效维护了社会稳定。各级政府依法接受人大及其常委会的监督，自觉接受人民政协和各民主党派、工商联、人民团体、无党派人士的民主监督。五年来，市人民政府共办理人大代表议案11件，建议和意见214件；办理政协提案791件。认真落实党风廉政建设责任制，深入开展纠风和执法检查专项治理，强化审计监督，从源头上预防治理腐败，查处了一批违法违纪案件，促进了勤政廉政。

各位代表，过去的五年，是改革开放以来我市经济社会发展最快的五年，是人民生活环境和精神面貌变化最大的五年，也是广大群众得到实惠最多的五年。

五年来，始终坚持紧扣发展这个第一要务，面对建市时间短、基础条件差、财政实力弱等不利因素，直面困难、不等不靠，在自治区党委、政府和市委的领导下，动员组织全市各族人民，开拓进取，奋力拼搏，开创了新市、穷市也能成大事、兴大业的良好工作局面，以卓有成效的工作，激发了全市人民的斗志，鼓舞了全市人民的士气。

五年来，始终坚持深入调查研究，紧密联系实际，准确把握市情，科学统揽工作大局，对不同地区、不同产业的发展实行统筹兼顾，分类指导，紧紧抓住了工作主动权，使全市经济和社会事业步入了健康快速的发展轨道。

五年来，始终坚持辨证地处理工业化、城市化、农业产业化三者的关系，坚定不移地把加快推进工业化进程作为全市经济发展的重要突破口，以工业化带动城市化和农业产业化，以城市化和农业产业化支持、促进工业化，形成了“三化”相辅相成、联动发展的良好运行态势。

五年来，始终坚持把维护稳定，加强团结作为经济和社会事业发展的基础，积极稳妥地处理民族宗教事务，深入细致地排查调处各类矛盾，开展形式多样的精神文明创建活动，加强了干群团结、民族团结、军政军民团结、中央及区属驻吴单位和地方的团结，为全市的经济发展形成了强大的合力。

各位代表，这些成绩和经验的取得，靠得是党中央、自治区的方针、政策的正确指引，凝聚着全市人民的辛劳和智慧，是方方面面关心支持、共同奋斗的结果。在此，我代表市人民政府，向全市广大工人、农民、知识分子和干部、驻吴部队、武警官兵、公安干警、中央及区属驻吴单位，向各位人大代表、政协委员，向各民主党派、各人民团体以及关心、支持吴忠发展的离退休老同志和社会各界人士，表示崇高的敬意和衷心的感谢！

回顾总结五年的工作，令人鼓舞，催人奋进，但我们更应清醒地认识到前进中的困难和问题。主要是：全市经济总量小，经济运行的质量和效益不高；工业化、城市化进程相对滞后，优势骨干企业集中度低，比较优势还没有显现；农业特色产业规模小、农产品市场竞争力不强、农民增收渠道单一、增长缓慢；城市建设规划滞后，经营、建设、管理城市的能力不强；财政自给能力弱，干部职工的收入相对偏低；就业再就业压力大，困难群众的生产生活问题还比较突出；投资环境有待于进一步改善，干部的思想观念有待于进一步转变，政府自身建设有待于进一步加强等等。对于这些问题，我们要采取更加有力的措施，认真加以解决。

二、今后五年奋斗目标和总体要求

党的十六大提出了全面建设小康社会的宏伟目标，党的十六届三中全会对进一步完善社会主义市场经济体制提出了明确要求，自治区党委、政府就宁夏今后一个时期的改革与发展已做出了全面深入的安排部署，为加快吴忠市各项事业的全面发展指明了前进的方向。从现在起到2008年，是我们实现建设经济强市，解决经济和社会生活中各种矛盾与困难的关键五年。面对区内外千帆竞发、百舸争流的竞争态势，肩负全市各族人民的期望与重托，我们必须树立强烈的忧患意识、责任意识和使命意识，一心一意谋发展，聚精会神搞建设，全力推进我市的改革开放和现代化事业。今后五年，政府工作总的指导思想与思路是：按照自治区党委和政府的总体部署，以邓小平理论和“三个代表”重要思想为指导，以全面建设小康社会总揽全局，认真贯彻落实党的十六大及十六届三中全会精神，坚持以人为本，树立全面、协调、可持续的发展观，始终不渝地实施工业强市战略、城市带动战略、项目带动战略与人才兴市战略，大力推进经济结构调整，推进工业化、城市化、农业产业化和对外开放，强力推进招商引资工作，大力发展非公有制经济与第三产业，着力营造产业优势，努力培植财源。促进物质文明、精神文明、政治文明共同进步，为实现吴忠经济社会的跨越式发展而努力奋斗。主要预期目标是：国内生产总值到2008年达到210亿元，年均增长12%以上，人均达到1万元。财政总收入达到12亿元，年均增长11.3%，其中市区两级财政收入3亿元，年均增长11%。全社会固定资产投资累计完成450亿元，年均增长15%。城镇居民人均可支配收入达到9000元，年均增长8%，农民人均纯收入达到3500元，年均增长6%。人口自然增长率控制在11‰以内。

为实现上述目标任务，我们必须坚持以国家产业政策、市场走向和资源优势作为谋划发展方向的基本依据，组织开展项目课题攻关大行动，切实把经济社会一个个宏观机遇变成我市项目开发的一个个亮点，把我市一个个资源优势转移成与国际国内市场良性接轨的一个个闪光点。把加强工业园区建设作为推动民资聚集、促进企业升级的主要载体，全面提高市县区各开发区、工农业园区的建设水平，本着项目成体系、布局成网络、设施成配套、发展成块状的目标，实施产业、企业、物业的“组团式”规则，不断增强其吸纳功能和承载能力，使吴忠市这块孕育着极大商机的热土尽快发展成新材料和农产品加工业的集合区、新兴物流业的商务区、依托黄河城市化的示范区。我们必须进一步聚合力量，强化措施，把加快发展非公有制经济作为引领全市广大人民群众创业的主战场，安置人民群众就业的主阵地，增加人民群众收入的主渠道，从拓展载体上多动脑筋，从打造平台上多下功夫，从加强对接上多办实事。加快发展非公有制经济的源泉在民众，出路在运作，途径在造势。按照抓有方向，干有目标，抓有把手，成有保障的原则，积极营造适应各类非公有制企业生长的小气候，大天地。我们必须聚全民之智、举全市之力、强力推进招商引资工作，通过再动员再部署，在发动程度、组织力度和推进强度上狠下功夫，全面掀起我市招商引资工作的新热潮。着力构建企业自主招商、机关干部驻点招商和社会群众引荐客商成功有奖的三位一体新格局；着力营造吴忠与外商“双赢、同发、共进”的亲商氛围。我们必须做好创新经济发展环境和净化投资环境的大文章，实行受理一个门、优惠一卡通、办事一站式、服务一条龙，让更多的投资者进得来、留得住、长得大、站得牢。通过建立各项相关规章制度帮助企业解决“围墙外的所有问题”，最大限度支持企业低门槛进入、低成本扩张、高效益运营。我们必须用典型引导群众，并将塑造典型的重心放在做大做强现有企业上，放在开发新的企业上，放在借助外资外力上。发挥政府职能，多做设置“航标”、消除“路障”的工作，帮助企业解决项目推进过程中的梗阻问题，多做银企对接的工作，帮助企业解决融资难的问题；多做“搭台”工作，组织企业与国内外大企业、大客商牵手联亲，力促品牌、资源、技术要素在合作中得到新的配置和升级。我们必须解放思想、转变观念、创新机制、大胆实践。加快发展的最大障碍是思想上的障碍，最大的差距是观念上的差距，我们只有立足于改变，否则没有任何出路。面对经济基础相对薄弱及科技、信息、人才等资源相对贫乏的市情，我们要做到一切从实际出发，牢固树立发展意识和危机意识，以招商引资、项目突破为经济工作的主抓手，不争论、不空谈，全力以赴抓招商，一心一意上项目。在发展道路上，要打破常规，大胆试，大胆闯，在“干”中前进，在“闯”中发展，让实践作结论，以实绩论英雄。以更为开放的思维，更为科学的态度，更为大胆的实践，努力把吴忠建设成为一个山川协调发展、区域经济特色鲜明、综合势力雄厚、民族团结、社会稳定、人民富裕、充满现代文明气息的宁夏经济强市。

做好今后五年的工作，我们要坚持六个始终不渝。

始终不渝地实施工业强市战略。把推进新型工业化放在经济工作的首要位置，列入各级政府工作的重要议事日程，作为一把手工程，不断改进工作方法，不断强化服务职能，为企业的发育成长提供良好的外部环境和有效的行政支持。继续推进企业改革，集中力量培育壮大优势特色产业，扶持发展更具活力的骨干企业和中小企业群，着力培育青铜峡区级新材料基地，实施夏进公司100万吨液态奶、美利纸业100万吨机制纸，中宁电厂、大坝电厂三期等大项目，使一批大企业进入全国同行业前列。力争到2008年，工业增加值达到115亿元，年均增长20%以上，使工业对国民经济的贡献率达到75%。以工业化带动城市化，进而带动整个经济发展。

始终不渝地实施城市带动战略。紧紧围绕《自治区城市化发展纲要》提出的目标要求，按照“依托黄河、北扩西移、吴青连接、相向发展”的思路，全力加快吴忠市区扩张建设，推进市区、金积、小坝连接，精心打造现代化滨河生态城市。全面加速县城和小城镇的开发建设，把中卫建成中等旅游城市，中宁实现老城区与石空镇区的依河融合，青铜峡实现与吴忠市区的跨河相连，把盐池、同心、红寺堡建成宁南山区更具活力的城市。宣和、叶升等一批中心集镇建设取得突破性进展。基本形成大中小城市及重点城镇梯度推进、协调发展的城市建设新格局，城市化率提高到45%，使人口、资源、要素、产业得到有效聚集，城市对经济社会发展的支撑、推动作用明显增强。

始终不渝地实施项目带动战略。完善创新工作机制，使招商引资工作经常化、制度化、规范化。以政府的主导作用带动社会的主体作用，鼓励引导干部职工、社会各界人士积极地融入市场竞争的大潮之中，敏锐地捕捉和争取每一个有助于吴忠发展的商机与项目，努力在全社会形成浓厚的招商引资氛围。配合实施中宁至营盘水、同心至沿川子、中宁至盐池高速公路、中太铁路，力争每年有几个大项目、有一批好项目在吴忠立地生根，开花结果。

始终不渝地实施人才强市战略。把人才作为推进经济社会跨越式发展的关键因素，紧紧抓住培养、吸引、用好人才三个环节，拓展人才工作的层面，努力创造人才充分发挥聪明才智的条件。树立不求为我所有，但求为我所用的新型人才观念，加强人才教育培训，培养造就高素质的城乡劳动者，大力加强以党政人才、企业经营管理人才和专业技术人才为主体的人才队伍建设，大力营造人才干事业，支持人才干成事业，帮助人才干好事业的社会环境，使各类人才创业有机会、干事有舞台、发展有空间，为全面建设小康社会提供坚实的人才保证和广泛的智力支持。强化教育对人才培养的基础作用，到2007年实现川区基本普及高中阶段教育、山区基本普及九年义务教育。

始终不渝地稳固农业的基础地位。统筹城乡经济协调发展，着力解决“三农”问题。用现代工业的理念和市场化手段发展农业，在加强耕地保护、确保粮食安全的同时，紧紧围绕农业增效和农民增收，加快农业经济结构战略性调整，提升产业化水平，真正把“三白一红一绿”特色产业做成强市富民的大产业。力争到2008年，全市奶牛饲养量达到15万头，羊饲养量达到600万只，畜牧业在农业总产值中的比重突破60%；发展枸杞18万亩，增加7万亩；酿酒葡萄10万亩，增加6.8万亩；优质饲草400万亩，增加220万亩。坚持不懈地推进农田水利基本建设，争取实施大柳树水利枢纽工程、扶贫扬黄续建工程、盐环扩灌等重点项目。加强生态环境治理，努力改善农业生产条件；不断加强封山禁牧、扶贫攻坚、劳务输出等工作，促进山川经济协调发展。

始终不渝地关心群众生活。把提高人民群众的生活水平、解决困难群众的生产生活问题，作为政府工作的出发点和落脚点，从践行“三个代表”重要思想的高度，满怀热忱、全心全意地抓好做实。高度重视就业和再就业工作，重点扶持“4050”人员实现再就业，新增城镇就业岗位6.2万个，实现劳务输出75万人次。密切关注困难群众和弱势群体的生产生活，继续做好“两个确保”，衔接好“三条保障线”，养老保险金征缴突破6亿元，失业保险金征缴突破8000万元，医疗保险参保人员突破15万人。加大行政执法监察力度，保障企业员工和农民工的合法权益。

各位代表，实现上述目标任务，我们还面临着许多困难和挑战。但是我们坚信，有自治区党委、政府和市委的坚强领导，有历届领导班子和广大干部群众打下的良好基础，有全市180多万各族人民发展吴忠、建设美好家园的崇高热情与强烈愿望，只要我们团结一致、奋力拼搏，只要我们勇于开拓，务实苦干，我们的目标就一定要实现，我们的目标一定能够实现！

三、二OO四年主要工作任务

2004年是新一届政府的开局之年，开好头、起好步，对于全面完成“十五”计划，顺利实现新一届政府任期目标至关重要。主要预期目标是：国内生产总值达到136亿元，增长13%，其中一、二、三产业分别增长4%、18.5%和9.5%；全社会固定资产投资完成90亿元，增长20%；地方财政收入力争完成6.28亿元，按可比口径计算增长13.8%，其中市区两级财政收入力争完成1.76亿元，增长13.9%；农民人均纯收入和城镇居民可支配收入分别达到2700元、6500元，增长5.8%和8%。为此，要突出抓好以下几个方面的重点工作：

(一)紧紧抓住第一要务，促进经济快速发展

以做大做强优势特色产业为重点，持续加快农业产业化进程，大力发展第三产业，不断壮大县域经济，努力提升全市经济的发展速度和综合实力。

加快培育产业优势，提升工业发展水平。继续把工业放在经济发展的突出位置，坚持以工业的大发展支撑市域经济的快速发展。一是集中力量培育优势特色产业。按照《吴忠市优势特色产业发展2004—2008年规划》要求，打破区域和城乡分割，打破工农界限，优化结构调整和产业布局，有效配置资源，整合生产要素，集中发展能源电力、新材料、造纸、奶、枸杞、葡萄酒、皮毛绒、建材、草畜等九大产业，使优势特色产业的集中度和生产规模都有大幅提升。力争全市优势特色产业新增产值40亿元。二是积极运作大项目、做强大企业。重点实施夏进公司5万吨液态奶扩建、青铜峡铝厂60万吨电解铝扩建、香山酒业3万吨“宁夏红”枸杞酒扩建、秦毅集团20万吨电解铝扩建、御马酒业10万吨葡萄酒、金昱元10万吨树脂和10万吨烧碱扩建、圣德华镁业和全世达镁业两个5万吨金属镁等项目，力争工业项目建设完成投资30亿元以上，新增产值14亿元。使骨干企业对工业产值的贡献达到80%左右，成为地方财力的重要来源。三是全力打造新的发展平台。紧紧围绕国家产业政策和自治区产业布局重点，立足各地的产业特色和资源优势，进一步完善工业园区发展的规划和政策措施，积极创造有利于企业发展的环境，不断加大招商引资力度，吸引项目进园区。积极争取自治区支持，尽快启动青铜峡区级新材料基地、同心韦州新材料基地建设，使园区和基地成为工业发展新的增长极。

积极推进产业化经营，努力增加农民收入。以农业增效、农民增收为核心，坚持用统筹发展的思路指导农村经济，用发展工业的理念发展农业，努力通过“三化”的互动互利使农村经济和农业效益得到大幅提升，农民收入有较快增长。一是深化结构调整，做强特色产业。围绕自治区“三个十”工程和优势产业带发展规划，深入调整农业产业结构和布局，集中力量，做大做强以“三白一红一绿”为主导的优势特色产业。突出发展畜牧业，特别是把奶产业作为强市富民的大产业来抓，加快西部乳都建设步伐，力争奶牛存栏再增1.5万头，达到8.1万头；继续实施盐池100万只滩羊、中卫3500万只鸡项目，培育发展全区最大的优质滩羊基地和西北最大的禽蛋加工销售基地；加快中卫、中宁、青铜峡生猪项目建设。推进枸杞标准化生产和精深加工，新增枸杞1.5万亩，达到13万亩；围绕建设西部第一林纸基地的目标，全力抓好50万亩速生林基地建设。同时，抓好中卫10万亩压砂西瓜、青铜峡5万亩葡萄、盐池100万亩中药材，盐池、同心、红寺堡三县区200万亩草产业基地建设，着力提高特色产业在农业产值和农民收入中的比重。大力培育优质无公害农产品，抓好中卫、青铜峡、利通区三个10万亩无公害蔬菜基地建设，力争全市20%以上的农产品实现无公害生产。二是推进产业化经营，做强龙头企业。加强龙头企业的现代化管理，大力支持夏进乳业、香山酒业、御马葡萄酒业，雄鹰皮草公司、法福来公司、天启薯业、绿海苜蓿产业公司等企业发展。争取新上万胜生物公司20万吨玉米淀粉扩建、汇丰公司2000吨牛羊肉加工、香山酒业2万吨枸杞饮料、天启薯业10万亩脱毒马铃薯收贮运销、太臻公司与绿宝公司两个5万吨苜蓿加工等农产品深加工项目，扩大龙头企业的生产和带动能力，促进农业和农民与市场的有效连接，依托龙头和基地增加效益和收入。三是加强农业基础设施建设，促进山川协调发展。继续加强农田水利基本建设，加强基本农田保护，确保粮食安全。配合自治区加快实施沙坡头水利枢纽工程、中卫南北干渠改造工程、青铜峡灌区节水改造工程、黄河综合整治工程。大力改善山区农业生产条件。抓好小流域综合治理、水土保持、农村人畜引水工程。切实加大扶贫攻坚力度，落实对口帮扶政策，全力实施智力扶贫、教育扶贫、项目扶贫、人财物扶贫，帮助山区群众尽快脱贫致富。坚持以生态建设为重点，集中发展草畜产业、生态农业、节水农业。全面巩固封山禁牧成果，扩大退耕还林还草面积。认真做好防汛防洪工作，确保山区经济较快增长。

大力发展第三产业，活跃城乡经济。充分发挥吴忠作为宁夏商埠重镇的优势，大力发展第三产业。把旅游业放在三产发展的优先地位，充分挖掘和整合旅游资源，充分运用市场机制，吸引社会力量参与旅游开发。在发挥自然景观优势，营造人文景观特色的同时，着力加强旅游软件建设，提高服务接待水平。着力打响“沙坡头”、青铜峡库区等旅游品牌，加快盐池哈巴湖、罗山生态旅游开发。大力发展现代商贸物流业，促进日用消费品、农资、农机、建材、摩托车、电器、农产品的流通贸易；积极发展餐饮等传统服务业。稳步发展房地产业。稳步推进各类市场的改造提升，发展专业市场和要素市场，培育发展经纪人队伍和流通组织，搞活商品流通。培育发展教育、卫生、文化、体育产业及社区经济，带活相关服务业。积极发展金融、保险、电信、通讯、中介服务等现代服务业，尽快形成新的商流、物流和信息流，聚集人气，活跃市场，拉动消费增长。使三产成为吸纳社会就业、促进经济增长、增加财政收入的重要力量。

发挥比较优势，壮大县域经济。坚持用市场经济的理念和方式，按照因地制宜、分类指导、山川协调发展的原则，突出特色，扬长避短，集中整合资源和生产要素，纵深推进工业化、城市化和农业产业化，着力培育重点产业和骨干企业，提升县域经济实力。切实加强对县域经济的考核，继续实施县域经济动态管理和激励机制，促进县市围绕经济发展、农民增收、财政增长、就业增加、项目争取理思路、求发展，努力形成你追我赶、不甘落后的竞争态势，力争使利通区、青铜峡、中卫、中宁四个川区县（市、区）在全区县市经济综合实力中位居前列，盐池、同心位次前移，红寺堡经济有较快增长。

(二)全面统筹城乡发展，大力推进城市化进程

坚持高起点规划，高标准建设，高效能管理，高水平经营，继续推动城市建设大干快上，着力增强城市的承载力、吸引力和亲和力。

高起点规划城市。围绕建成滨河生态城市的定位，尽快完成吴忠市区总体规划修编，完善城市功能分区规划及道路、供排水、燃气、绿化等专业规划，编制主要街区、重点地段的控制性详细规划和城郊村庄改造规划。进一步修编完善县(市、区)城市规划。增强规划的科学性、前瞻性、指导性。加强规划监管，严格实行规划审批行政责任追究制度，严格执行城市规划法规，维护规划的权威性和严肃性。

高标准建设城市。市区建设以拓展新区，拉大城市框架为重点，投资25亿元，实施建设项目120个，完成建筑面积150万平方米，扩展市区面积10平方公里。集中力量实施黄河吴忠市区过境段综合整治工程，加快滨河大道及6条连线道路建设，新建、改造吴灵西路、西一环路、新村南街、西环路等12条城市道路。抓好吴忠汽车站迁建、人民会堂、新闻中心、公安侦察指挥中心等25个重点项目建设。实施城市供水管网改造、供热扩容及热计量改造工程，开工建设秦渠南岸集中供热、污水处理等项目。完成拆迁60万平方米，建成住宅53万平方米。重点抓好西花园小区、华苑小区、古城庄点等7个城市住宅小区和城郊小康村示范点建设。坚持把城市绿化摆上重要位置，实施拆建让绿、环城植绿、拆墙透绿、沿街铺绿工程，切实抓好城市生态公园、黄河生态绿化、城市主干道绿化、街头小坪及小区绿化，使城市人均公共绿地面积增加2平方米，城市绿化覆盖率提高4个百分点。县(市、区)城市建设继续坚持突出特色与定位，集中资源、资金和项目，扩张规模，完善功能，提升品位。加快中卫滨河新区开发、青铜峡小坝东区建设、中宁县城南扩北连、盐池城区拓展及环城造林、同心城区改造扩建、红寺堡城区建设，力争完成投资35亿元以上。把集镇建设作为统筹城乡发展的重要突破口，集中扶持发展金积、叶升、宣和、石空、韦州、惠安堡等中心集镇，增强小城镇在城乡经济社会发展体系中的纽带和牵引作用。

高效能管理城市。积极推行“两级政府、三级管理、四级网络”的城市管理体制，逐步建立责权利相统一、工作重心下移的长效管理机制。加强社区建设，充分发挥社区在城市管理中的基础作用。加大城市管理综合执法力度，强化市容、环卫、交通管理，加强对城市脏、乱、差及城乡结合部卫生死角等问题的专项治理，加快实施重要路段、街区广场、窗口地区的美化亮化工程，塑造市容整洁、环境优美、秩序井然的城市新形象。

高水平经营城市。充分发挥市场机制的作用，注重运用市场手段经营城市。政府依法垄断土地一级市场，放开搞活二级市场，坚持土地规划、征用、开发、供应、管理“五个统一”，加大土地储备、出让、招商力度，加强土地收益的征缴和管理，最大限度获取土地增值收益。加快市政公益事业改革步伐，有效盘活城市资产，大力推进城市供水、供热、公交、广告等基础设施的市场化和社会化，积极探索公益设施特许经营权拍卖、有偿使用和经营权转让，多渠道筹措和增加建设资金，推动城市建设走滚动发展、良性循环的路子。高度重视和解决城市化进程中的矛盾和问题，切实维护稳定和广大人民群众的合法权益。

(三)深入推进各项改革，致力发展非公有制经济

按照完善社会主义市场经济体制的要求，全面深化各项改革，着力推进招商引资和非公有制经济的发展，增强全市经济发展的活力与后劲。

全面推进各项配套改革。深化国有企业改革，促进国有资产战略性改组，强化国有资产监督管理，依托优势企业盘活存量资产，促进资源和生产要素向有潜力、有活力的产业、企业汇聚流动。不断创新企业内部管理机制，完善现代企业法人治理结构。按照依法、自愿、有偿的原则，建立市场化的土地承包经营权流转机制。坚持政事公开，推进事业单位的分类改革和乡镇撤并后的配套改革。加快公共财政建设，深化预算编制、国库集中收付及“收支两条线”管理改革，全面推行政府采购制度。加快教育、卫生等社会事业改革，建立多种形式共同发展的社会事业服务网络。

高度重视招商引资工作。牢固树立抓发展必须抓项目，抓经济就要抓招商的理念，把2004年作为全市的项目招商年，将招商引资和项目建设放在抓好经济工作的首位，实施环境招商、资源招商、亲缘招商，着力营造全民招商的浓厚氛围。紧紧抓住国家继续实施积极财政政策的机遇，努力争取水利、交通、文教、卫生、旅游、城市基础设施建设和农业产业化等方面的投资项目，狠抓一批能够带动全市经济发展的好项目、新项目、大项目。实行招商引资目标责任制和重大项目领导负责制，组织企业及相关部门走出去，多形式、多渠道招商引资；强化激励机制，重奖对招商引资有突出贡献的单位和个人，鼓励社会各个方面紧紧围绕我市资源优势和特色产业，加大外引内联，吸纳外商投资和社会投资。

大力发展非公有制经济。充分发挥非公有制经济在促进经济增长、扩大就业和活跃市场等方面的重要作用，加速县域经济民营化步伐。降低民营企业注册条件，放宽民间资本的市场准入领域，鼓励和引导民营企业以参股、控股、兼并、收购等方式参与国有企业的改革、重组和改造。进一步拓宽非公有制经济融资渠道，坚持抓大扶强，培育发展一批上规模、上水平，经济效益好、市场竞争力强的民营企业。依法加强监督和管理，依法保护民营企业的合法权益，促进非公有制经济健康发展。力争非公有制经济产值达到122亿元，增长20%。

(四)坚持执政为民，努力提高人民生活水平

始终把提高人民生活水平作为一切工作的出发点和归宿，关注民生，执政为民，努力为群众解难题、办实事。

积极扩大就业和再就业。把扩大就业放在经济社会发展更加突出的位置，坚持劳动者自主择业、市场调节就业、政府促进就业的方针，按照以发展增加就业，以调整促进就业，以改革带动就业，以开放推动就业的思路，进一步完善再就业工作机制，建立健全再就业服务体系，认真落实促进就业和再就业的各项政策措施，强化就业指导和技能培训，不断拓宽就业空间，力争年内新增就业岗位12000个，安排“4050”人员就业1000人，城镇登记失业率控制在4%以内。

强化社会保障工作。建立健全同经济发展水平相适应的社会保障体系。以巩固“两个确保”为基础，加强“三条保障线”衔接工作。继续完善城镇职工基本养老保险、失业保险和基本医疗保险制度，尽快建立工伤保险制度。抓好城市低保扩面和提标工作，确保应保尽保。发展社会福利和优抚安置等各项保障事业，切实加强城市流浪乞讨人员的管理工作，确保困难弱势群体的基本生活。建立和完善以基本生活救助为重点，以医疗、教育、住房、法律、项目等专项救助为辅助的农村社会救助体系，保障农民群众的基本生活权益。力争年内养老保险金征缴突破亿元，医疗保险参保人数突破10万人，失业保险金征缴突破1500万元，工伤保险参保人数突破3万人。

做大做强劳务经济。充分发挥政府在劳务经济发展中的主导作用，建立和完善长效机制，把推进“三化”同转移农村剩余劳动力紧密结合起来，建立健全农村劳动力培训组织体系，兴建吴忠市农民工就业培训基地，多形式、多层次提高农民工素质。引导农民合理有序地进城务工经商，拓宽失地农民向非农产业转移的渠道。同时积极兴办各类农副产品加工、储藏、运销企业，拓展农村就业空间，力争实现劳务输出15万人。

全面提升社区服务功能。以完善社区服务体系为重点，大力发展社会救助和便民利民服务，积极探索社区服务市场化、社会化的运行机制。全面实施“星光计划”，进一步改善社区居委会办公条件。发展社区医疗卫生服务，繁荣社区文化，加强社区警务建设。充分发挥各类社区居民自治组织的作用，推动社区向自我管理、自我服务、自我监督的目标迈进。

(五)大力发展各项事业，促进社会全面进步

充分发挥科技在经济社会发展中的支撑作用，加大科技攻关、科技示范、科技成果推广力度。继续实施科技特派员创业行动，加快吴忠国家农业科技园区建设步伐。全面发展教育事业，全力以赴打好山区“普九”攻坚战。进一步整合教育资源，加强优质学校建设。大力发展职业教育，积极发展民族教育，争取我市有一批中小学进入全区“百标工程”。积极鼓励支持社会力量兴办教育，促进民办教育健康发展。继续抓好人口与计划生育工作，全面推行计划生育优质服务，确保全市计划生育率达到85%以上，人口出生率控制在18‰以内。加强公共卫生设施建设，建成吴忠市疾控中心和市医院传染病区。建立应对突发公共卫生事件的医疗救助体系。广泛开展形式多样的群众性文体活动，不断丰富和活跃城乡文化生活。严格执行安全生产责任制，大力消除事故隐患，确保人民生命财产安全。切实加大环境保护执法力度，清理整顿不法排污，严格控制新污染源产生。全面落实党的宗教政策，依法管理宗教事务。加强国防教育，完善国防动员体制。广泛开展优军优属、拥政爱民活动，进一步密切军政军民关系。关心支持老龄、残疾人事业。切实做好外事、侨务等工作。

(六)加强精神文明和民主法制建设，创造良好的社会环境

坚持两手抓，两手都要硬，不断加强精神文明和民主法制建设，为经济与社会事业的发展提供有力支撑。

加强社会主义道德建设。深入开展邓小平理论和“三个代表”重要思想教育，认真落实《公民道德建设实施纲要》，大力倡导“爱国守法、明礼诚信、团结友善、勤俭自强、敬业奉献”的基本道德规范，继续深入开展文明单位、文明社区、文明村及五好家庭等群众性精神文明创建活动，积极倡导良好的社会公德、职业道德和家庭美德，提高社会文明程度。

大力推进依法治市进程。依法接受人大及其常委会的法律监督和人民政协的民主监督，认真办好人大议案、建议和政协提案。建立和完善重大决策的规则和程序。发挥民主党派、工商联、无党派人士参政议政作用和工会、共青团、妇联组织的桥梁纽带作用，广泛听取群众的意见和要求。健全基层自治组织，完善村民自治和居民自治，完善村务公开和厂务公开，完善职工代表大会和其他形式的企业民主管理制度。加强政府法制工作，坚持依法行政，从严治政，努力提高依法行政水平。继续抓好“四五”普法教育，提高全民法律素质。

创新经济发展环境。坚持把创新经济发展环境当作促进发展的助推器。强化人人都是发展环境，事事都是发展大局的意识，在加强硬环境建设的同时，着力围绕政府职能转变，坚决排除一切有碍发展的政策性、体制性障碍。切实优化政府管理经济与社会的职能，强化对经济的宏观调节、市场监管和公共服务。坚决杜绝部门乱罚款、乱摊派、乱收费和“吃、拿、卡、要”行为。大力整顿和规范市场经济秩序，严厉打击各种假冒伪劣行为和不正当竞争，强化企业及经营者的信用教育，深入开展“信用吴忠”创建活动，不断提高人文素质，营造优良的创业环境和人居环境。

全力维护社会稳定。全面落实社会治安综合治理的各项措施，完善“打防控管一体化”的治安防范网络，大力整治突出的社会治安问题，依法严厉打击各类刑事和经济犯罪，防范、打击暴力恐怖活动，坚决扫除“黄、赌、毒”等危害社会的丑恶现象，加大对“法轮功”邪教组织的打击力度，有效预防和减少犯罪，使社会治安发案率明显下降。加强信访工作，畅通信访渠道，认真做好矛盾纠纷排查调处工作，努力把各种矛盾解决在基层和萌芽状态，确保社会稳定。

(七)认真践行“三个代表”重要思想，努力建设为民、务实、高效、清廉的政府

按照建立“学习型、法治型、服务型、廉政型、诚信型”政府的要求，切实加强政府自身建设。

强化教育，提高素质。切实加强公务员培训，组织广大公务员认真学习政治、经济、文化、科技、法律、WTO规则等方面的新知识和新理论，拓宽新视野，开拓新思维，不断提高公务员队伍的整体素质，增强促进发展、爱岗敬业、务实苦干的自觉性，提高配合大局、服务经济、解决问题、做好实际工作的能力，培养和造就一支讲政治、讲奉献、守纪律、会干事的高素质公务员队伍。

转变职能，提高效率。深化行政体制改革，切实改变政府管理经济和社会事务的方式，继续推进政企分开、政事分开和政社分开，把政府职能转移到经济调节、市场监督、社会管理和公共服务上来。精减审批事项，简化审批程序，推行“一站式”服务，拓展政务大厅的办理事项和范围，规范操作制度，严肃工作纪律，提高办事效率，切实提高服务社会、服务群众、服务客商的能力。逐步形成行为规范、运转协调、办事公开、务实高效的行政管理体制，推动政府工作全面提速。

拓宽渠道，透明决策。积极探索建立和拓宽联系基层、联系群众的渠道，密切与群众的血肉联系。通过“市长专线”、“环境创新投诉专线”等途径，体察群众疾苦，倾听群众呼声、意见和要求，自觉接受群众的批评与监督。认真落实信访工作目标责任制，把群众来信来访作为了解下情的主要渠道，主动采取措施，努力缓解矛盾，妥善解决问题。坚持政务公开，做到决策内容、决策程序、决策依据向社会公开，保障群众的知情权、参与权和监督权。建立完善重大行政决策合法性、可行性论证机制，提高行政决策科学化、民主化水平，不断提高政府的公信力。

健全机制，廉洁勤政。认真落实领导干部、公务员廉洁从政的各项规定，建立和完善思想教育、制度规范、监督制约、查案惩处、廉政责任机制，提高拒腐防变能力。严格监管土地批租、工程招标、产权交易、人事调配等环节，严格领导干部经济责任审计和“收支两条线”管理，严肃财经纪律。依法重点查办领导干部违法违纪、执法人员贪赃枉法等案件，真正树立起政府机关工作人员的良好形象。

改进作风，执政为民。大力弘扬求真务实、讲求实效、真抓实干、雷厉风行的工作作风，努力克服官僚主义和形式主义的东西。强化政府部门和领导的工作责任，建立发展县域经济、改善人民生活和招商引资的目标管理责任制，严格考核，狠抓落实。经常深入基层，调查研究，认真解决人民群众关心的热点难点问题。坚持从群众最不满意的事情抓起，从群众最困难的事情帮起，从群众最期望的事情做起，努力以新的形象、新的作风、新的业绩向全市人民交上一份合格的答卷。

各位代表！

我市今后五年和今年政府工作的总体要求和目标任务已经明确，全市人民对我们寄予了殷切希望和重托，展望现代化建设的大好前程，我们激情满怀；面对改革发展稳定的艰巨任务，我们责任重大。让我们高举邓小平理论伟大旗帜，全面贯彻“三个代表”重要思想，在市委的坚强领导下，积极进取，发奋图强，以硬举措实践硬道理、以新思路谋求新发展，为吴忠经济和社会全面进步作出更大贡献！
